# Wavelength Discrimination in Drosophila Suggests a Role of Rhodopsin 1 in Color Vision

**DOI:** 10.1371/journal.pone.0155728

**Published:** 2016-06-03

**Authors:** Christian Garbers, Thomas Wachtler

**Affiliations:** Department Biologie II, Ludwig-Maximilians-Universität München, Planegg-Martinsried, Germany and Bernstein Center for Computational Neuroscience Munich, Munich, Germany; University of Western Australia, AUSTRALIA

## Abstract

Among the five photoreceptor opsins in the eye of Drosophila, Rhodopsin 1 (Rh1) is expressed in the six outer photoreceptors. In a previous study that combined behavioral genetics with computational modeling, we demonstrated that flies can use the signals from Rh1 for color vision. Here, we provide an in-depth computational analysis of wildtype Drosophila wavelength discrimination specifically considering the consequences of different choices of computations in the preprocessing of the behavioral data. The results support the conclusion that Drosophila wavelength discrimination behavior can best be explained by a contribution of Rh1. These findings are corroborated by results of an information-theoretical analysis that shows that Rh1 provides information for discrimination of natural reflectance spectra.

## Introduction

Color vision is widespread across the animal kingdom. It has been demonstrated in many insect species, including the fruit fly *Drosophila melanogaster*[1]. The sensory basis for color vision is the presence of photoreceptor types with different spectral sensitivities. Five different photoreceptor types exist in the ommatidial eye of Drosophila. Their respective photosensitive opsins are called rhodopsin 1 (Rh1), rhodopsin 3 (Rh3), rhodopsin 4 (Rh4), rhodopsin 5 (Rh5), and rhodopsin 6 (Rh6). [2, 3] The ommatidia can be grouped into two types. In the so-called pale ommatidia the inner receptors cell R7 (R7p) expresses Rh3, while the R8 (R8p) cell, positioned below R7, express Rh5. In the so called yellow ommatidia R7 (R7y) expresses Rh4, while R8 (R8y) expresses Rh6 [4–7]. Furthermore, in the ommatidia that span the dorsal third of the retina, Rh3 is co-expressed within the cells that normally express only Rh4 (R7y) [8]. In the dorsal most rows of cells both inner receptors express Rh3 [9, 10]. Until recently the common assumption was that Rh1 does not contribute to color vision [11, 12]. Furthermore, the outer receptor cells are equipped with an sensitizing pigment, which makes them additionally receptive in the UV [13–15].

In a recent study that combined results from computational modeling, electrophysiology, and behavioral genetics, we have shown that fruit flies are able to discriminate stimuli based on chromatic differences even when only signals originating from Rh1 and a single other opsin are present [16]. This implied that Rh1 can be used for color vision in the fruit fly. The modeling results were based on published data on wavelength discrimination derived in behavioral experiments. Because there is some freedom in the derivation of a quantitative estimate of wavelength discriminability, the method of analysis might have an influence on the outcome. Therefore, we performed an in depth computational investigation on the role and impact of Rh1 signals in wildtype Drosophila wavelength discrimination, and we analyzed in detail whether changes in the assumptions underlying the derivation of behavioral wavelength discrimination data would influence the results.

While it has been shown that dichromatic flies were able to discriminate narrow-band stimuli using signals from Rh1 [16], the influence of Rh1 on wildtype Drosophila color vision is still an open question. In general, the usefulness of having five receptors for color vision could be taken into question. For human color vision, based on reflectance data from Munsell chips and the observation that reflectance spectra are band-limited functions, it has been argued that a finite linear model of 6–12 parameters should be sufficient to completely reconstruct reflectance spectra from “color signals” [17, 18]. This can be interpreted as an upper bound for the maximum number of receptor types that would make sense to code for color [17, 18]. However, the number of photoreceptors that would practically be beneficial has been estimated to be lower [18]. In general, Vorobyev [19] analyzed the accuracy of reconstruction of fruit and flower reflectance under realistic levels of receptor noise. He found that for an animal with a visual system extending into the UV, pentachromacy did not provide a significant benefit over tetrachromacy. It is therefore questionable whether the signals from Rh1 would actually be informative.

We therefore analyzed natural reflectance spectra from a large database [20]. We determined the information content across wavelengths and show that indeed in the range around 500 nm, where we have found that Rh1 is necessary to explain wildtype wavelength discrimination, information about spectra identity is available from Rh1. In an additional theoretical analysis of the natural reflectance spectra in the frequency domain we determined the number of receptor types that would suffice to acceptably approximate the data. We analyzed, based on mutual information [21], the amount of information in the signals from Rh1 and analyzed how much of this information is already transmitted by the other opsin.

## Methods

### Quantifying wavelength discrimination

**Fig 1:**
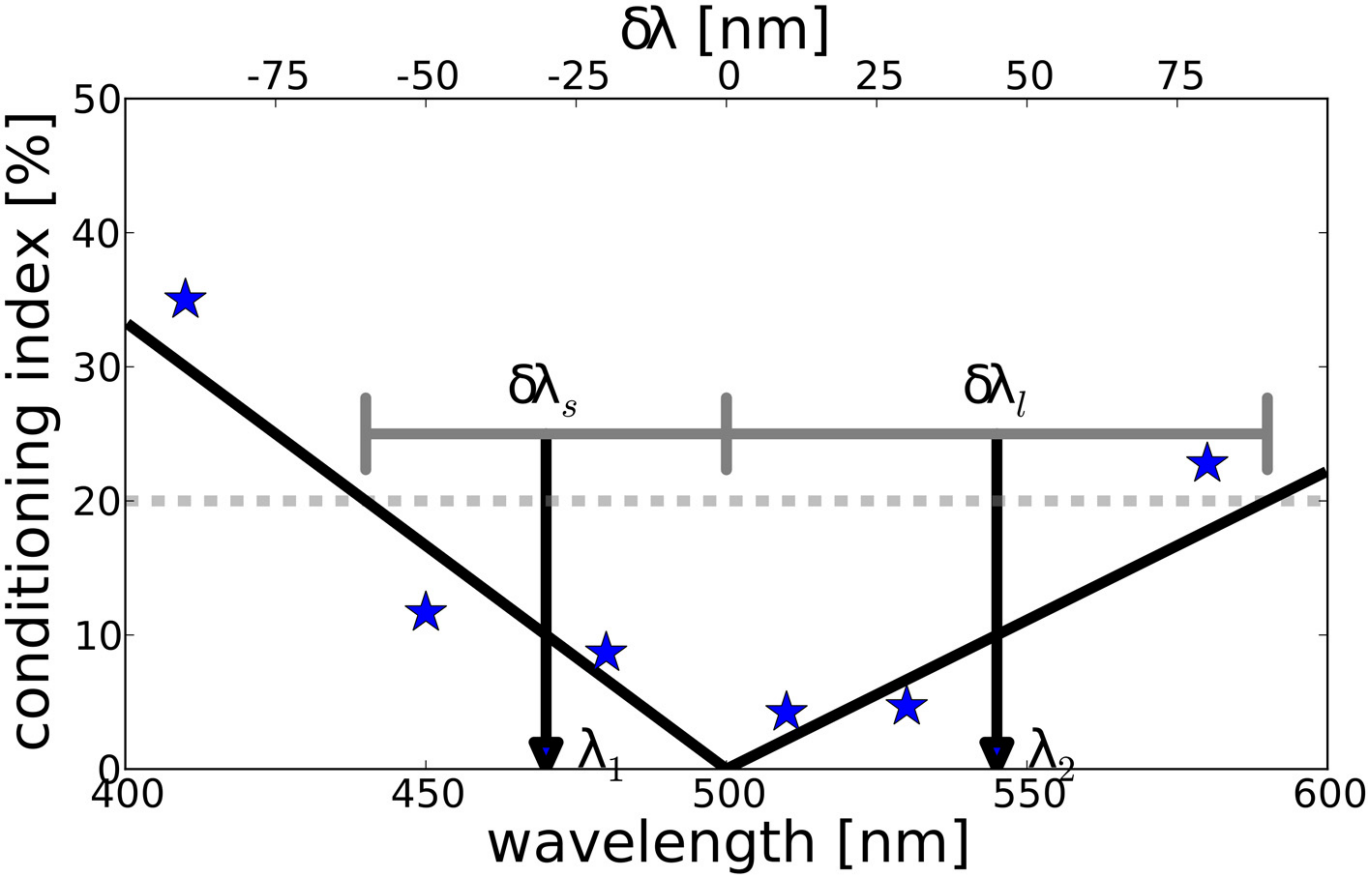
Example plot of conditioning index data. Animals had been trained to discriminate a reference stimulus of 500 nm from stimuli of other wavelengths as indicated on the horizontal axis. The vertical axis shows for each pair how many animals, above chance, gave the correct response. The black solid line shows a linear interpolation of the data points. The dashed line indicates an (arbitrarily chosen) threshold of 20%. *δλ*_*i*_ are the ranges between the reference wavelength and the intersection between the threshold and the interpolation of the data, as indicated by the gray horizontal lines. The midpoints of these ranges, *λ*_1_ and *λ*_2_, are the virtual reference wavelengths as used to derive the wavelength discrimination function [24].

The ability to discriminate stimuli varying in wavelength has been quantified by deriving so-called *δλ* functions [22]. A *δλ* function indicates the minimal change in wavelength that is necessary, at a certain reference wavelength, for an animal to discriminate a stimulus from the reference wavelength. In insects and other animals, such quantitative estimates on wavelength discrimination have been derived from discrimination learning experiments [23]. Animals were conditioned to choose a certain wavelength above another wavelength, and the animals’ performance was quantified by determining how many animals, or how often an animal, was able to discriminate the stimuli. The corresponding probability is called a conditioning index [23]. The procedure is repeated for several wavelength pairs, resulting in a conditioning index function (see Fig 1).

To derive a quantitative *δλ* estimate from conditioning index functions, a threshold *T* is defined (Fig 1). The amount of wavelength change necessary to reach this threshold is then defined as the discriminability at that reference wavelength. By analyzing several conditioning index curves in this way, an estimate of the *δλ* function was derived by determining the *δλ* for which the discrimination conditioning function *L*_*λ*_0__(*δλ*) reached the threshold T,

$$L_{\lambda_{0}}\left(\delta \lambda \right)=T.$$ (1)

This threshold is arbitrarily chosen and different values have been used. Therefore the derived estimates constitute only a lower bound of the animal’s ability to discriminate light stimuli by wavelength [24].

Eq 1 typically has two solutions, one for longer (*δλ*_*l*_) and one for shorter (*δλ*_*s*_) wavelengths. To derive a unique discrimination value per reference wavelength, several strategies have been used. If the two solutions are not too different a method is to take the mean of the two values [25].

$$\frac{\delta \lambda_{l}+\delta \lambda_{s}}{2}.$$

If the solutions are very different this results in information loss and a less precise estimate. To preserve more information from the conditioning curves into the *δλ* estimates, Von Helversen [23] used a different approach. He kept the two values but derived new virtual reference wavelengths for them by taking the midpoints of the intervals [*λ*_0_, *λ*_0_ + *δλ*_*l*_] and [*λ*_0_ − *δλ*_*s*_, *λ*_0_] (see Fig 1 for an illustration) denoting *δλ*_*l*_ or *δλ*_*s*_, respectively, as the wavelength discrimination values for the two virtual reference wavelengths,

$$\lambda_{1}=\lambda_{0}-\frac{\delta \lambda_{l}}{2}$$ (2)

towards shorter wavelengths, and

$$\lambda_{2}=\lambda_{0}+\frac{\delta \lambda_{s}}{2}$$ (3)

towards longer wavelengths. We will call this the split-reference transformation.

This approach circumvents the problem of having two discrimination values per reference by creating two virtual references. It therefore results in more data points for the wavelength discrimination function. As both virtual reference wavelengths depend directly on measured *δλ*s (see Eqs 2 and 3), which have error bars, the positions of the new references are also uncertain. Therefore, these *δλ* values have errors in x and y [24].

### Modeling wavelength discrimination

To determine wavelength discrimination functions, we used an approach based on the method of Vorobyev and Osorio [26], who modeled spectral sensitivity functions of opponent combinations of receptor responses and calculated the distances between stimuli in the space of such opponent responses, taking into account the estimated noise in the photoreceptors. We did not make any assumptions about the noise and more generally asked whether there is a way, to linearly combine the opponent channels such that the result would fit to the data.

Let Δ*q*_*i*_(*λ*) be the signal difference that two stimuli evoke in receptor i at wavelength *λ*. Then for two receptor types 1 and 2 the signal in a neuronal channel k that combines these two receptor signals opponently is

$$S_{k}^{2}\left(\lambda \right)={\left(\Delta q_{1}\left(\lambda \right)-\Delta q_{2}\left(\lambda \right)\right)}^{2}.$$ (4)

The Euclidean distance in a space with a basis formed by several of such opponencies can be used to predict spectral sensitivity [26]. In the case of Drosophila with five rhodopsins, there are ten different opponent combinations. From this pool of potential opponent channels we calculated relative spectral sensitivity thresholds for visual systems combining information from several of these channels by summation over the signals from the n respective channels

$$S^{2}\left(\lambda \right)=\sum_{k=1}^{n}w_{k}S_{k}^{2}(\lambda ),$$ (5)

where *w*_*k*_ is a vector of weights that scales the opponent channels relative to each other.

Δ*q*_*i*_(*λ*) corresponds to the slope of the spectral sensitivity of the ith receptor at wavelength *λ*.

From *S*^2^(*λ*) we calculate wavelength discrimination by taking the inverse

$$D\left(\lambda \right)=\sqrt{\frac{1}{S^{2}\left(\lambda \right)}}.$$ (6)

We fitted wavelength discrimination functions for different visual systems by adapting *w*_*k*_ to minimize the squared distance between model and data from Hernandez de Salomon and Spatz [24]. Fitting was performed with a variant of the Levenberg-Marquart algorithm implemented in the Python programming language [27]. We fitted models for different hypothetical visual systems. We started with models with a single opponent channel, then proceeded to fit all possible combinations of two opponent channels, then three, and so forth. In this way we fitted all possible combination up to eight combined mechanism. Including more channels would have reduced the number of degrees of freedom below 1. However, the systems with large numbers of channels always yielded poor fits with p below 0.05.

Opponent channels were derived from published Drosophila spectral sensitivities and had sensitivity maxima at 478 nm (Rh1), 345 nm (Rh3), 375 nm (Rh4), 437 nm (Rh5) and 508 nm (Rh6), respectively (see Fig 5 in [14]). Spectral sensitivities were scaled to peak at unity.

To quantify goodness of fit between a model and the behavioral data on wavelength discrimination we used the *χ*^2^ statistic

$$\chi^{2}=\sum_{i=1}^{n}\frac{{\left(x_{i}-y_{i}\right)}^{2}}{\sigma_{i}^{2}},$$ (7)

where *x*_*i*_ are the observed discrimination values and *y*_*i*_ predictions from the model. *σ*_*i*_ is the standard deviation of the data. The error on the wavelength axis (see above) was transformed into a discrimination error by estimating the impact of the wavelength uncertainty with respect to the current model estimate. For a given datapoint we calculated the maximum discrimination uncertainty that the associated wavelength error would have by deriving the maximum discrimination change that the current model estimate had in a range corresponding to the given error around the data point. For the wavelengths *λ*_*i*_ with empirical data on wavelength discrimination

$$E\left(\eta \right)=D\left(\lambda_{i}\right)-D(\eta ).$$ (8)

is the difference in discrimination between the wavelength *λ*_*i*_ and *η*. By taking the maximum of *E*(*η*) in a range given by the error in the wavelength Δ*_i_* we derived an upper bound for the discrimination uncertainty due to wavelength uncertainty,

$$\max_{\eta \in [\lambda_{i}-\Delta_{i},\lambda_{i}+\Delta_{i}]}\frac{1}{2}\left|E\left(\eta \right)\right|$$ (9)

By adding this additional error to the discrimination error, the *χ*^2^ statistic took uncertainties in both dimension into account. This is a rather liberal strategy which was used to be inclusive towards models without Rh1.

The *χ*^2^ value, which is a weighted sum of squared errors, does not take the number of fitted parameters into account. Under the assumption that the underlying random variable is independent and standard normal, the *χ*^2^ values follow a *χ*^2^ distribution derived for a number of degrees of freedom. From this *χ*^2^ distribution we can directly get the likelihood of a given *χ*^2^ value. We derived likelihoods for all fits. Only fits that could not be excluded under the null hypotheses (p-value > 0.05) that the data had been generated from the model, were analyzed for receptor contributions as described below.

We quantified which receptor types contributed to the discrimination in models for visual systems that fitted the data (p > 0.05) by calculating the weight of a certain receptor in all models relative to the sum of weights for all receptors in all models. In the same way, we quantified the contribution of the ten opponent channels.

To analyze the influence of the chosen transformation from conditioning function to *δλ* function, we determined the *δλ*_*s*_, *δλ*_*l*_, and *λ*_0_ values used in Hernandez de Salomon and Spatz [24] and re-derived wavelength discrimination functions, using the mean transforms introduced above.

### Analysis of natural reflectance spectra

To determine the potential contribution of Rh1 to Drosophila color vision, we quantified the amount of available information as a function of wavelength by calculating the differential entropy of the spectra dataset in 1 nm intervals. Differential entropy extends the idea of Shannon entropy, a measure of average surprise of a random variable, to continuous probability distributions. In our case, it indicates how informative signal variation at a given wavelength is with respect to spectra identity. The higher the value the more information can be gained by observing the value at that wavelength.

The differential entropy of a random variable *x* with probability density *f*(*x*) is defined as

$$H\left(x\right)=-\int_{X}p(x)\;\log(p(x))dx$$ (10)

We calculated the differential entropy for each wavelength using Gaussian kernel density estimation as implemented in scipy [27].

To estimate the number of receptors that would theoretically be useful to account for natural color variability, we calculated the power spectral density of the natural reflectance spectra under D65 illumination using discrete Fourier transform. The power spectrum of a reflectance spectrum *x*(*λ*) describes how the variance of the spectrum is distributed over the frequency components into which it may be decomposed. Note that frequency in this case does not refer to the frequency of the electromagnetic wave but rather to the abscissa of the Fourier transform of the spectral reflectance curve and is therefore measured in cycles per wavelength. If the power spectrum is band limited, i.e. above a certain frequency practically no power is left, then such band limited function can very accurately be approximated by a linear model of a few parameters. The number of parameters is determined by the Whittaker, Kotelnikow and Shannon sampling theorem [28]. In the case of visual systems it determines the number of receptors useful to approximate the spectra in a given visual range (300 nm–550 nm).

The critical question, however, is which receptors are best suited to extract such information and, for the case of Rh1, how much non-redundant information can the fly gain by integrating the information from Rh1. To quantify this, we determined the amount of information about the spectral composition of the environment contained in the photoreceptor signals. We calculated the mutual information between the photoreceptor outputs $\vec{O}$ as determined by the spectral sensitivities and the spectral inputs $\vec{W}$, using the established method of of Lewis and Zhaoping [21]. We did the calculations using the Drosophila spectral sensitivities, normalized to unit area, and one more principal component as well as a a larger set of reflectance spectra. The spectra used were from an online database [20] and were mainly reflectances of various species of flowers. Apart from that, the method was as described by Lewis and Zhaoping [21]. We calculated the information assuming equal noise proportional to the square root of the signal in all receptors. This corresponds to a situation of normal lighting [21].

Mutual information measures how much information about a random variable *Y* is obtained by observing another variable *X*. In our case it indicates how much uncertainty about color inputs ($\vec{W}$) is removed by observing the photoreceptor outputs ($\vec{O}$). Formally this is defined as

$$I=\int_{p}p\left(\vec{O},\vec{W}\right){\text{log}}_{2}\left[\frac{p(\vec{O},\vec{W})}{p\left(\vec{O}\right)p\left(\vec{W}\right)}\right]d\vec{W}d\vec{O},$$ (11)

where $p(\vec{O},\vec{W})$ are the joint probability distributions of $\vec{O}$ and $\vec{W}$, and $p(\vec{O})$ and $p(\vec{W})$ their respective marginals. We estimated $p(\vec{W})$ from the natural reflectance spectra by means of principal component analysis on the spectra dataset. We represented each spectrum by its power in the first four principal components, which capture 95% of the variance in the data. We then fitted a four-dimensional truncated Gaussian to this dataset and hence derived $p(\vec{W})$. Truncation was done under the constraint that mean and variance were fixed, that is, the variance of the fitted function was the same as the variance of the data. To arrive at $p(\vec{O},\vec{W})$, we calculated $p(\vec{O}|\vec{W})$ assuming that receptor signals for a given reflectance $\vec{W}$ (calculated with respect to the spectral sensitivity of the receptor and the derived principal components) vary due to Gaussian noise. With $p(\vec{O}|\vec{W})$ known, $p(\vec{O},\vec{W})$ is simply $p\left(\vec{O}|\vec{W}\right)p\left(\vec{W}\right)$ and $p(\vec{O})$ can be calculated as $p\left(\vec{O}\right)=\int p\left(\vec{O},\vec{W}\right)d\vec{W}$. Numerical integration was performed on Tesla K80 GPU Accelerators using PyCuda [29] and custom written compute kernels. Detail’s on the methods can be found in the original publication [21]. Parameters used to calculate the information can be found in Tables 1 and 2.

**Table 1:** Factors describing the relation between principal component and spectral sensitivity. The values are the inner product between spectral sensitivities and eigenfuctions of the spectra dataset calculated for D65 illumination (see [21] Eq 4).

	Rh1	Rh3	Rh4	Rh5	Rh6
PC 1	5.58	0.944	1.65	4.35	5.92
PC 2	3.38	-3.59	-3.53	1.34	3.24
PC 3	-5.17	-0.654	-2.83	-6.94	-1.84
PC 4	-3.04	-0.67	-0.164	-0.614	-4.70

**Table 2:** Factors describing the probability density of the spectral sensitivities. First two column give the mean and variance of the first four principal components for the set of reflectances. The third column gives the fraction of the total variance explained by the corresponding principal component. The fourth column gives the associated eigenvalues.

	*μ*	*σ*^2^	Var. explained	Eigenvalue
PC 1	0	5.05	0.52	3.822e+05
PC 2	0	0.501	0.19	1.386e+05
PC 3	0	1.29	0.17	1.270e+05
PC 4	0	0.75	0.06	4.691e+04

## Results

**Fig 2:**
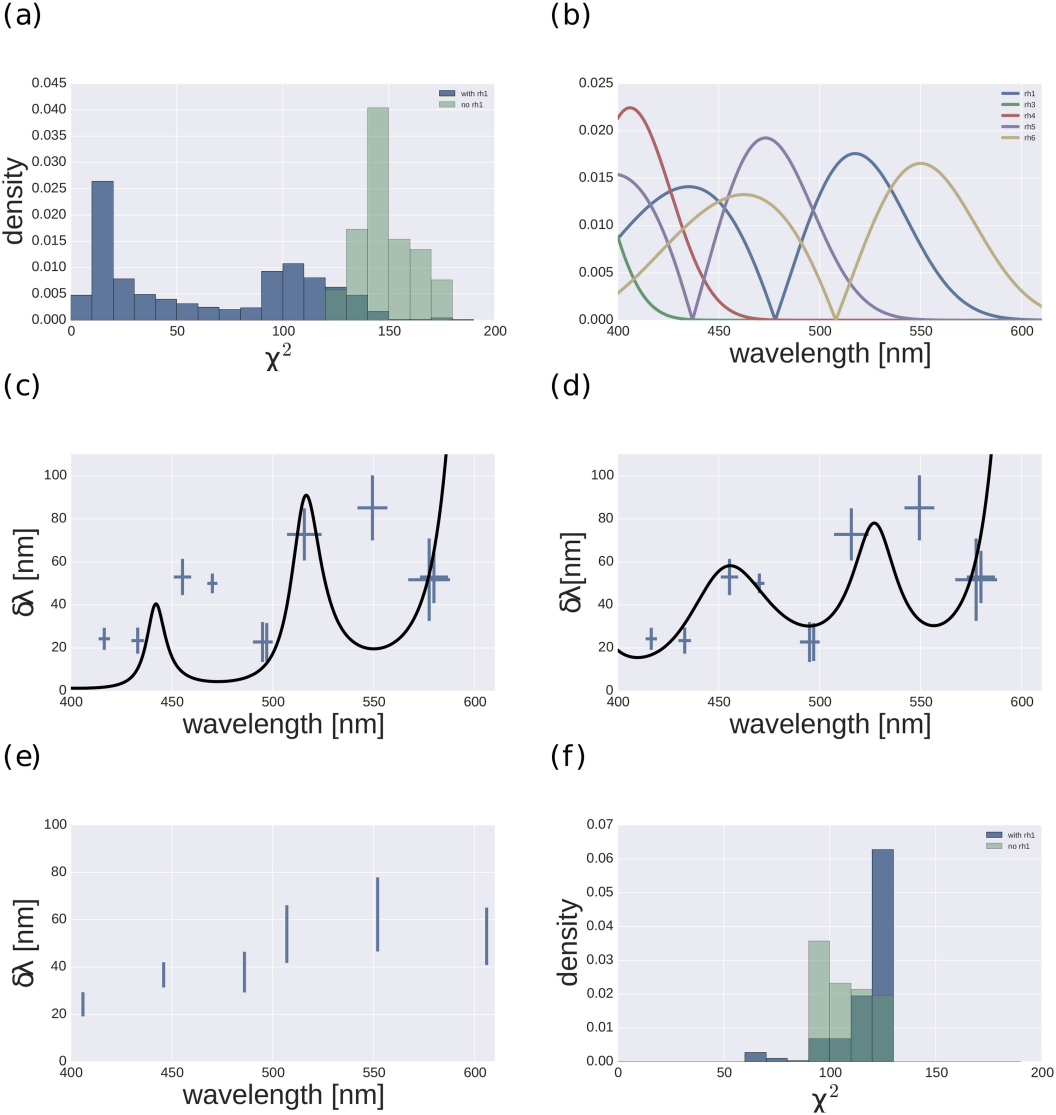
Fit statistic over all possible models and the best fitting models. (a) Histograms of *χ*^2^ values for all fits of models without Rh1 (green) and with Rh1 (blue). (b) Absolute slopes of Drosophila opsins in the visual range. (c),(d) Best fitting models without Rh1 (i.e. Rh4-Rh6, Rh5-Rh3; weights:609.9 177.1) and with Rh1 (Rh1-Rh6, Rh4-Rh6; weights 100.5, 84.4). (e) Mean-transformed data. (f) Histogram of *χ*^2^ values for fits of the mean-transformed data for models without Rh1 (green) and with Rh1 (blue).

Generally, models including Rh1 fitted the data better than models without Rh1 (see Fig 2). Fig 2a shows the distribution of the *χ*^2^ values calculated over all fitted models with and without a contribution of Rh1. Models without Rh1 generally gave poor fits (best fit p-value below 0.001, Fig 2c), while a subset of the models with Rh1 explained the data well (best fit p-value of 0.17, see Fig 2d). Statistics for the best fitting models disregarding one of the opsin types can be found in Table 3.

**Table 3:** Best fitting models when one opsin type is removed. The first column indicates the missing opsin type. The second column indicates the mechanism and the third column the associated weights. The fourth column indicate the associated p values.

Missing Opsin	Mechanisms	Weights	p
Rh1	Rh3-Rh5, Rh4-Rh6	610, 177	0.0000
Rh3	Rh1-Rh6, Rh4-Rh6	101, 85	0.17
Rh4	Rh3-Rh6, Rh1-Rh6	125, 219	0.0016
Rh5	Rh1-Rh6, Rh4-Rh6	101, 85	0.17
Rh6	Rh1-Rh3, Rh1-Rh4, Rh3-Rh4, Rh4-Rh5	71, 70, 61, 48	0.0000

### Contribution of Rh1

The most prominent difference between models with and without Rh1 was the ability of the Rh1 models to fit the steep increase in discriminability between 470 nm and 500 nm that is evident in the data. This increase in discrimination cannot be explained without a contribution of Rh1, as Rh1 is the only opsin with increasing slope in that region (Fig 2b) and such an increase in the slope of the spectral sensitivity is a prerequisite for a better wavelength discrimination.

**Fig 3:**
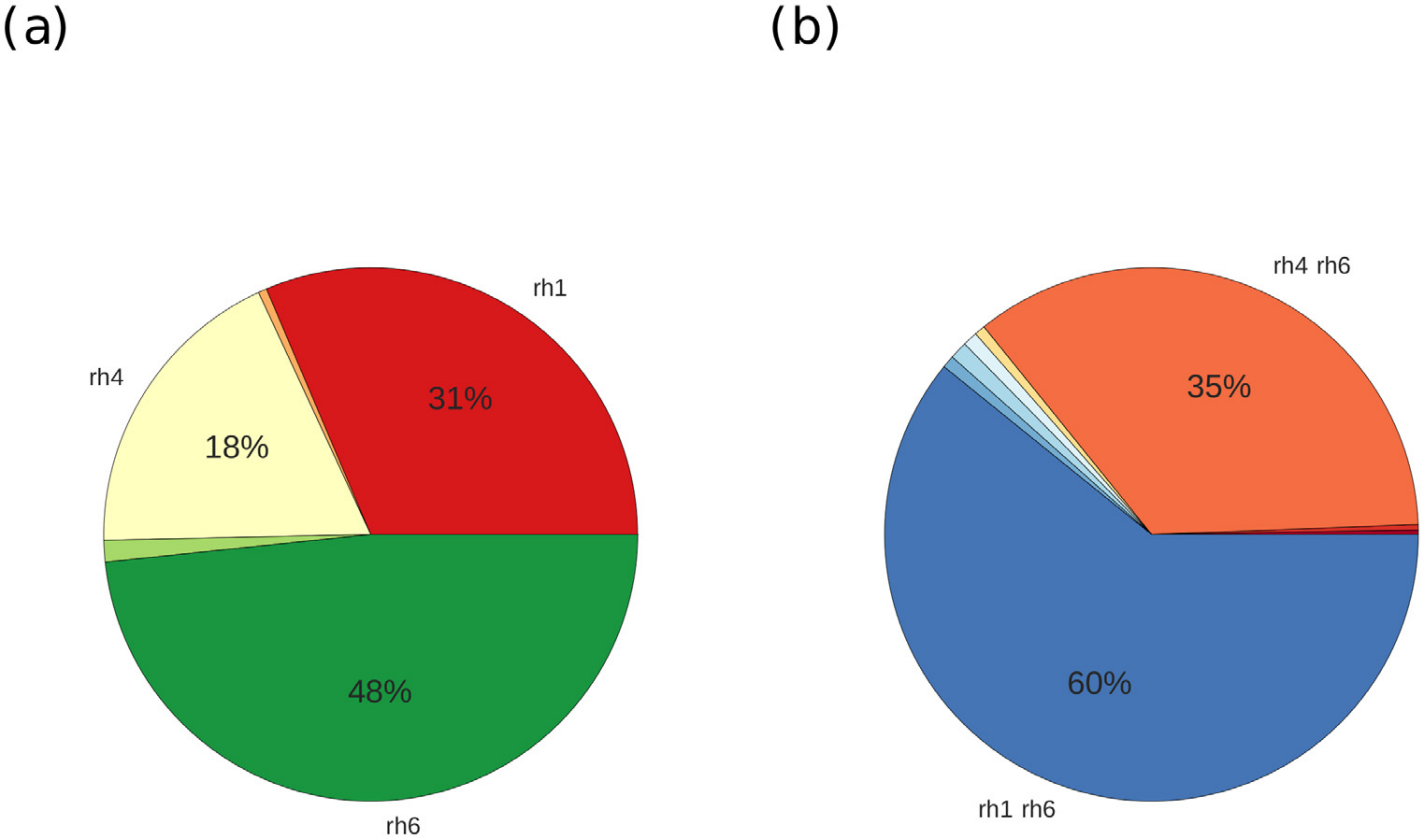
Contributions of receptors and opponent mechanisms to the model fits (p>0.05). (a) Relative contribution of each receptor, measured by the total sum of weights over all fits. (b) Relative contributions of each opponent mechanism; For readability only contributions of 3% or more are labeled.

In the models that fit well (p > 0.05), Rh1 was the opsin that contributed second-most to the fits. Only Rh6 contributed more (Fig 3a). Among the opponent channels, Rh1-Rh6 contributed most to models that gave good fits (Fig 3b). Together, Rh1-Rh6 and Rh4-Rh6 made up more than two thirds of the overall contribution.

### Alternate data transformation

While the data from the split-reference tranformation showed multiple wavelength regions of good and poor discrimination, the mean transformation led to data that indicated one wavelength region of good discrimination, for short wavelengths, and one region of less good discrimination, for long wavelengths (Fig 2e). Furthermore, the number of data points was reduced to six (see Methods). While the best fitting model for the data from the alternate transformation was also a model with Rh1, in general all models had to be rejected (p< 0.001) and the clear difference in fit quality, which was apparent for the data from the original transformation, disappeared. This means that no model was able to explain the data from the alternate transformation.

### Encoding of natural spectra

**Fig 4:**
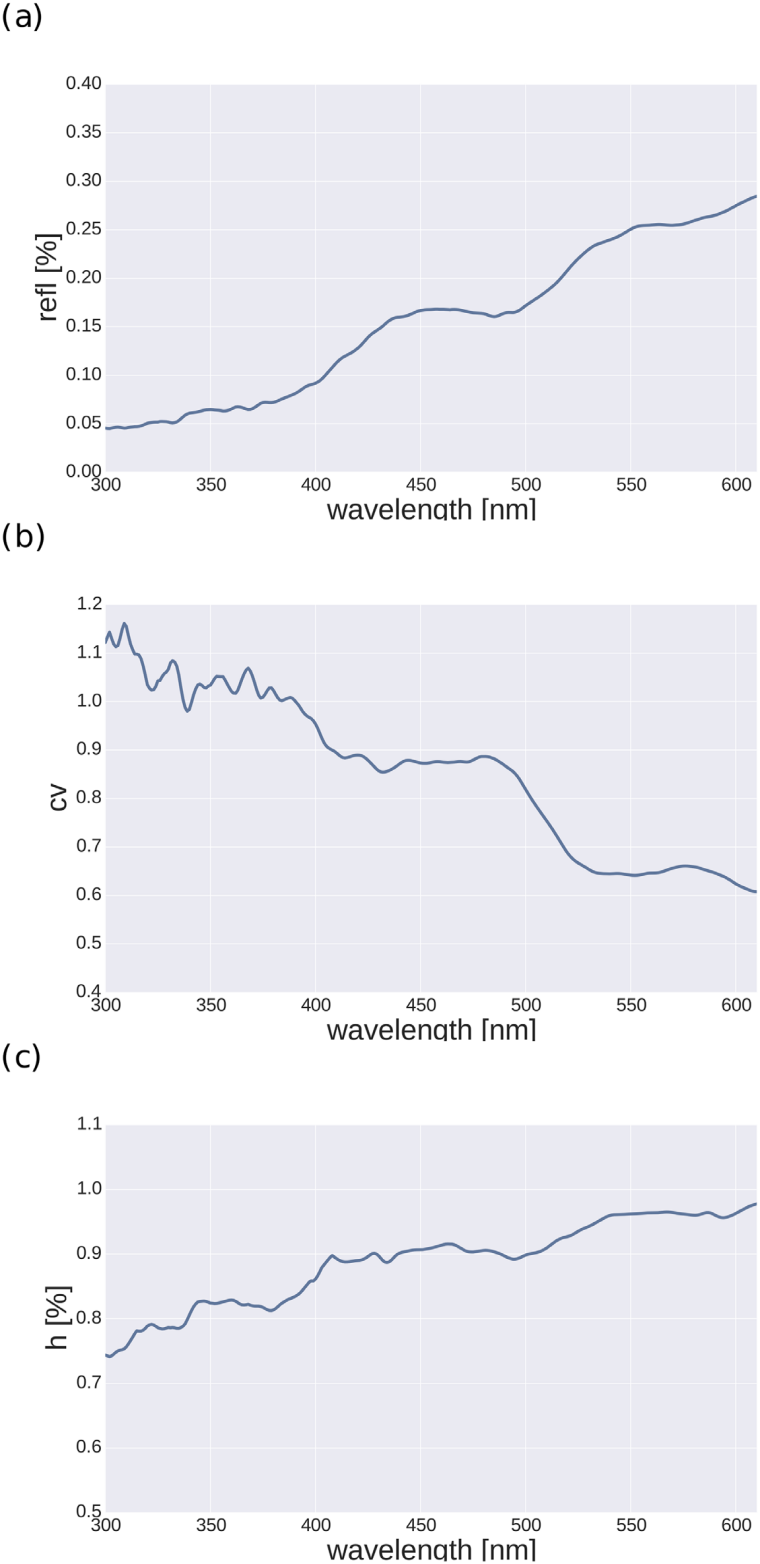
Natural spectra statistics. (a) Average over all spectra in the FRED database. (b) Coefficient of variation as function of wavelength. (c) Differential entropy. All plots show values calculated for each wavelength bin separately.

Fig 4 shows the statistics of the natural spectra. The average spectrum is maximally reflective in the long wavelength range. Likewise, the differential entropy indicates that the long wavelength range is most informative, with a steep decline in information below 550 nm. In the range between 500 nm-400 nm it reaches a rather stable plateau. This plateau is followed by another decline to another plateau below 380 nm. Interestingly, in a range above 490 nm, where Rh1 is the most sensitive opsin (see. Fig 2b), the differential entropy starts to rise, and it is this very area where Drosophila wavelength discrimination is best [24].

**Fig 5:**
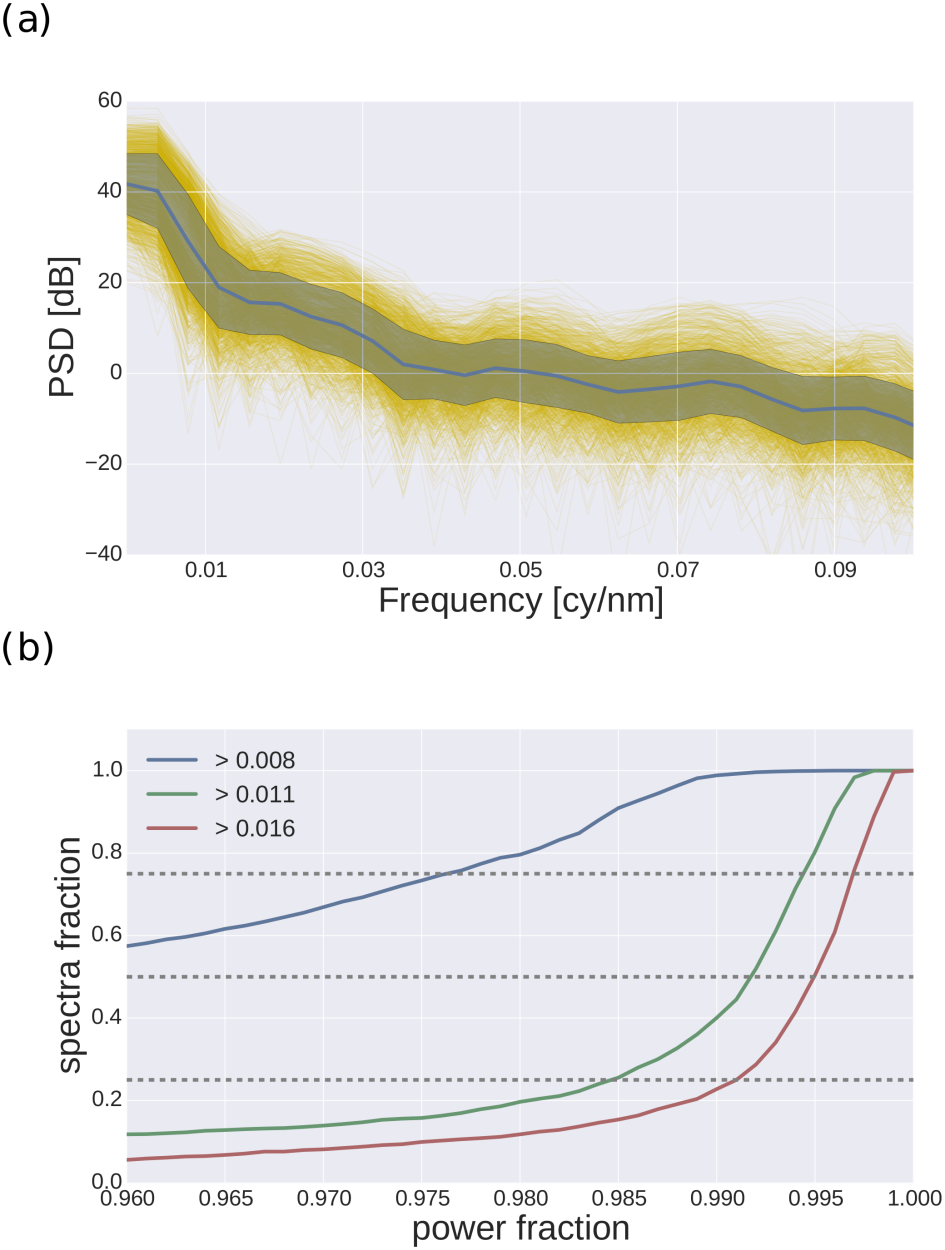
Power spectral density of natural spectra. (a) Average psd calculated over all spectra (blue line, the shaded area indicates standard deviation). Individual psd are plotted as thin yellow lines. The abscissa indicates spectral frequency in cycles per nanometer. The ordinate indicates power measured in dB. (b) Fraction of spectra (ordinate) that have a cumulative power fraction below a certain value (abscissa). Values are plotted for cut-off frequencies of 0.008 (blue), 0.011 (green), and 0.016 (red). Dotted lines indicate quartiles.

The average power spectral density of natural spectra can be seen in Fig 5a. Most power is in the low frequencies, and power spectral density shows a rapid decline with frequency, which becomes slower around 0.01 cy/nm. Fig 5a shows the cumulative distribution of spectra for a given power fraction, calculated for three cut-off frequencies. At a cut-off frequency of 0.011 cy/nm, most of the spectra have already lost 98.5% or more of their power. This is in line with previous findings [18], but here we used a larger set of different reflectance spectra. It confirms that natural reflectance spectra are approximately band limited with a cut-off frequency of 0.01 cy/nm. Thus, it is sufficient to sample changes in reflectance that have a cycle length of 100 nm. Considering the sampling theorem [28] and by assuming a visual range of 300 nm-550 nm, we can conclude that five receptors, evenly spaced 50 nm apart from each other (eg., with peaks at 325 nm, 375 nm, 425 nm, 475 nm, 525 nm) would perfectly sample the natural data variability.

**Fig 6:**
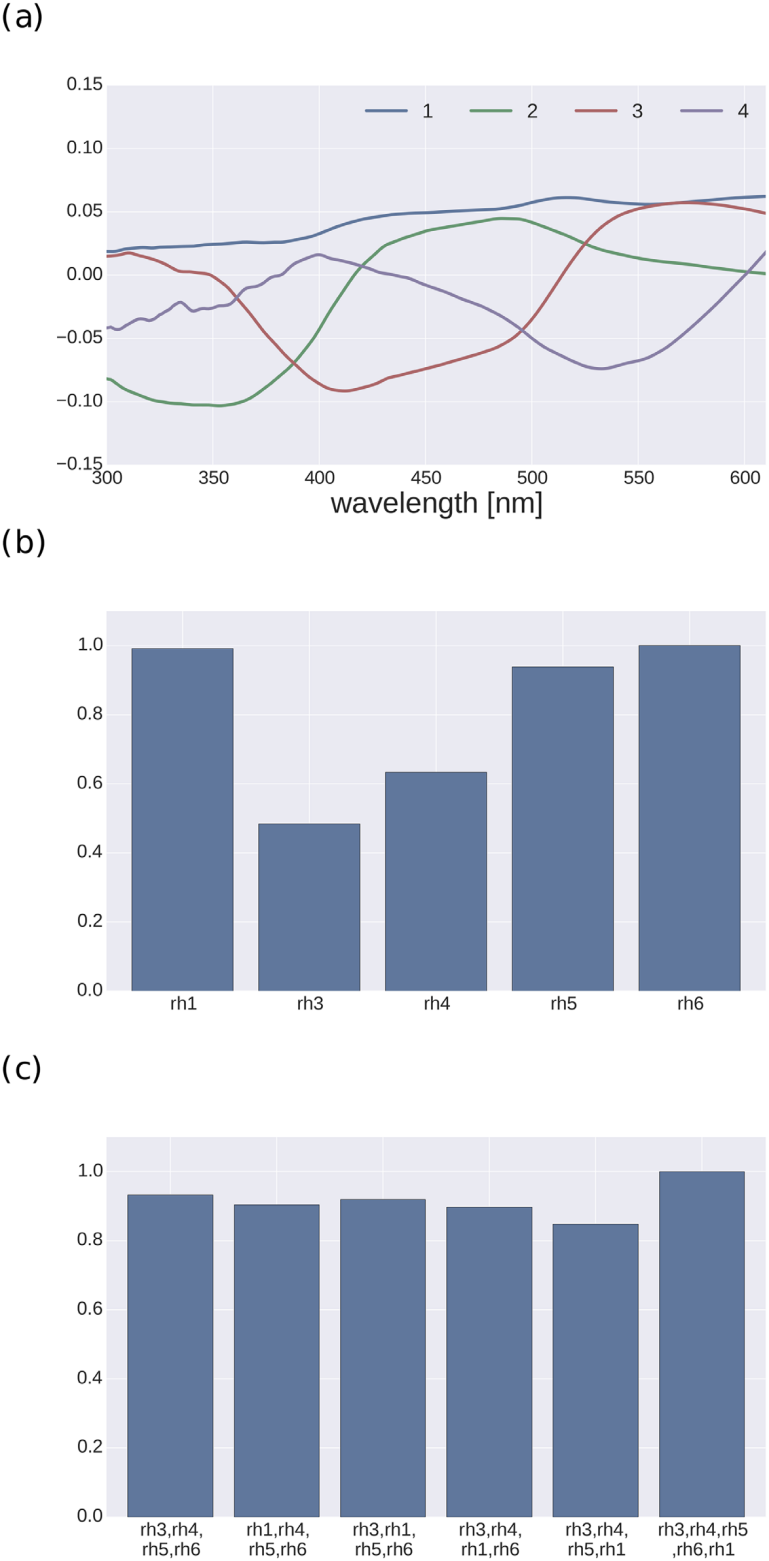
Mutual information. (a) First four principal components calculated over all spectra. Curves indicate unnormalized raw PCA values as a function of wavelength. (b) Mutual information between the individual receptors and the spectra. (c) Mutual information between the five possible systems with four receptors and the system with five receptors. Values are reported as fraction of the maximum [21].

The mutual information in the five receptor types is shown in Fig 6b. The Rh6 opsin is most informative, directly followed by Rh1 and Rh5. The two UV receptor types are only half as informative as their longer wavelength companions. Of the five possible four-receptor combinations, the traditional system without Rh1 is actually most informative, while for the other combinations the informational content is higher when substituting lower wavelength opsins with Rh1. Finally, the addition of the Rh1 opsin to the traditional system, leading to a five receptor system, is only 7% more informative. On average, moving from three to four receptors systems adds 17% of information; from two to three receptors 21% (data not shown).

## Discussion

In a previous study we focused on models that, considered as being implied by the retinal architecture, included comparisons between inner ommatidial receptors only (Rh3-Rh5 or Rh4-Rh6). Here we provide a more in-depth analysis on the role of the outer receptors with Rh1. We determined the best fitting opponent models that either included or did not include Rh1. The well fitting models all made use of spectral information from Rh1. Furthermore, the opsin that contributed most to the good fits was Rh6, directly followed by Rh1. Additionally we found that the Rh1-Rh6 opponency together with the Rh6-Rh4 opponency explained most of the data. The reason for this can be found in the spectral profiles of the opponent mechanisms. The only mechanism that had a maximum in the slope near 500 nm, where the data indicate good wavelength discrimination in the fly, was the Rh1-Rh6 opponency. For shorter wavelengths, around 470 nm, the behavioral data suggested a lower discrimination, requiring a lower slope, as exhibited by the Rh1-Rh6 mechanism. Another increase in discrimination at even shorter wavelengths is also supported by the Rh1-Rh6 opponency. Above 500 nm, the data indicated a sharp decline in discriminability. This decline was supported by a decline in the slope of the Rh4-Rh6 opponency, the mechanism which might also well contribute to the increase in discriminability between 470 nm and 400 nm. Thus, a combination of the two opponent mechanisms, Rh4-Rh6 and Rh1-Rh6 already explains the data quite well.

Two aspects of the behavioral data may have led to an overestimation of the role of Rh1. First, there are two data points near 500 nm, which amplifies the requirement of a mechanism that explains the data in this region. However, repeating the analysis with one of these data points excluded did not lead to qualitatively different results. Furthermore, the absence of data in the UV range clearly downplays the role of the two UV opsins (Rh3, Rh4). Nevertheless, while quantitatively the weight of Rh1 in the model fits might overestimate its role, the qualitative argument holds that the increase in discrimination performance between 470 and 500 nm can only be explained with a contribution of Rh1. Concerning the role of the other opsins it is surprising that Rh5 did not contribute to the best fitting models. Among the models with a p value above 0.05 there were models with significant contribution from Rh5. However, the majority of well fitting models did not include Rh5. Considering the nature and sparsity of the dataset (see also below) as well as the data from dichromatic flies [16], it should not be concluded that Rh5 does not contribute to color discrimination in wildtype Drosophila. Nevertheless, the data used here is best explained by a model that uses only Rh1, Rh4 and Rh6.

Only models using Rh1 yielded fits with p values above 0.05. However, even the best fitting model had a p value of only 0.17. This low value was mainly due to the poor fit to the data point at 550 nm. If this data point is excluded, the fit quality rises to a value of 0.75. It is important to point out that the predicted good wavelength discrimination at 550 nm that can be found in the best fitting models is a direct consequence of the spectral sensitivity of the Rh6 opsin (see Fig 2b). The Rh6 slope peaks at 550 nm and therefore models including Rh6 necessarily predict better discrimination at 550 nm than for longer wavelengths. In their original publication, Hernandez de Salomon and Spatz [24] pointed out that errors in the data increased considerably above 500 nm, and in particular that the value at 578 nm is not significantly different from the value at 550 nm. At wavelengths above 500 nm the overlap between spectral sensitivities, a necessary prerequisite for color discrimination, is low, and moreover, the two curves have slopes of same sign. Together with the low overall sensitivity in this wavelength range [24] it seems feasible that the stimuli were not properly matched for brightness and that therefore values above 500 nm are unreliable. Overall the goodness of fit was not very high for any of the models. Comparable analyses of wavelength discrimination are rare [30], and it is not clear whether better fits could be expected at all.

In our modeling paradigm, opponent channels are insensitive to intensity changes of broadband light, but for monochromatic stimuli, this is not strictly speaking the case. We nevertheless assumed that non-opponent mechanisms do not play a role for wavelength discrimination. This assumption has been shown to be valid in bees [31], but so far not in Drosophila. While the available data for Drosophila are not rich enough to apply the approach used in bees, we tried to fit purely non-opponent models, i.e., models that combine receptor signals additively, including approaches based on the envelope of the spectral sensitivity curves. None of these models yielded fits that were as good as those with opponent models (data not shown). For the more interesting case of a mixture of non-opponent and opponent mechanisms, we introduced all possible non-opponent combinations of two receptors to the mechanisms used for fitting. We performed fits with all possible models combining up to five mechanisms. As was the case for models comprised exclusively of opponent mechanisms, none of the mixed models without Rh1 provided a good fit. Non-opponent mechanisms did not contribute strongly to the best fitting models. For example, the best fitting mixed model had a p value of 0.15 and was a combination of the two chromatic mechanisms that also gave the best fit in the chromatic case (Rh1-Rh6, Rh4-Rh6) and one non-opponent mechanism (Rh4+Rh5). The weights of the three mechanisms were 98, 75 and 6, respectively, indicating a very low contribution of the non-opponent mechanism. In general, non-opponent mechanisms contributed less than 5% of the weight to the models giving good fits, indicating that spectral discrimination in Drosophila is mainly based on opponent signals.

In cases where noise is proportional to the signal, it has been shown that the logarithm of the receptor signals can be a better choice to model spectral data [32]. Several models in the literature also had a nonlinear component [32–34], however linear approaches also have been shown to yield reliable estimates [35]. We therefore performed an analysis assuming logarithmic receptor signals. We found that no model (neither with Rh1 nor without Rh1) gave acceptable fits. The best fit was by a model that combined the two channels Rh1-Rh6 and Rh3-Rh6. However, even with this model, goodness of fit (p< 0.001) was orders of magnitude below the fits of the linear models.

Relating the slope of the spectral sensitivities of the photoreceptors to wavelength discrimination implies that *δλ* is small enough so that the slope can be taken as constant. The values reported in [24] can be as high as 80 nm, a value for which it seems unlikely that this assumption is valid. However, there is reason to interpret these values as relative as opposed to absolute. First, it would be inconsistent to have a discrimination threshold of 80 nm at one wavelength, and 50 nm away a threshold of 20 nm, as is the case in this dataset. As explained above, the values derived by the method of Hernandez de Salomon and Spatz depends on an arbitrarily chosen threshold and provides only a lower estimate of the ability of the flies to discriminate wavelength [24]. Furthermore, the data were obtained in experiments where the behavior of a population of flies was measured. In this paradigm, flies that did not learn the task would have decreased the resulting value of wavelength discrimination. Studies of wavelength discrimination in other insects that reported much lower discrimination thresholds [12, 23] had been performed on individual animals. It can be assumed that in those studies, animals that did not learn the task had been excluded

Other studies on wavelength discrimination have specifically included assumptions about background illumination [30]. We therefore tested whether such a modification would improve the fits and tested the model with the assumption of a background illumination with the spectrum of either a Tungsten lamp (see [30]) or the standard daylight D65. However, in both cases the fit quality decreased compared to the model without assumption of a specific background illumination.

It would be of high value to have a larger dataset on Drosophila color discrimination, ideally measured directly at wavelengths where *δλ* is comparable for shorter and longer wavelength. With respect to our main finding, a denser sampling of the region between 450 nm and 500 nm could provide a critical test of a contribution of Rh1. It would also be interesting to test animals with stimuli that are metameric with respect to all but one opsin type, as has been done in primates [36] using broadband stimuli. This type of stimulation, however, requires very precise knowledge of the shape of the spectral sensitivities, which is currently not available for Drosophila. Furthermore, it might be hard to achieve high enough contrasts, especially for photoreceptors with similar or broad spectral sensitivities. Testing individual flies [12] in combination with probabilistic choice modeling [37, 38] and the derivation of psychometric functions which account for lapse rates and biases [38–40] might help further to reduce arbitrariness and noise in the estimates.

In general, the mean-data transform results in a discrimination function that is less complex than the function obtained with the split-reference transformation. Besides the reduction in the number of data points, the discrimination function indicates one region of good discrimination in the short wavelength range and one region of poor discrimination in the long wavelength range, with a rather steep transition. While in this case the best fitting model was a model with Rh1 as well, all fits were poor. Thus, the mean-data transformation leads to *δλ* estimates that can hardly be explained by a linear combination of the rhodopsin spectral sensitivity slopes. This clearly argues in favor of using the split-reference transformation to derive wavelength discrimination functions at least in cases where the conditioning index functions are rather asymmetric with respect to the reference wavelength.

One caveat of the split-reference transformation is that a change of the criterion level to calculate the *δλ* values not only changes the discriminability values. Because the virtual reference wavelengths depend on the derived discriminabilities, both x and y values of the data points change with changing criterion levels. This effect is more severe for datapoints where discrimination is poor than for points with good discrimination. If discrimination is good, the slope of the conditioning index curves is high, implying that a change in the criterion level, defined on the y-axis, leads to small changes in the reference wavelength. Our main finding is mainly due to two points with good discrimination near 500 nm, and it is therefore rather robust against reasonable changes in the criterion level.

Considering natural reflectance spectra, the conditioned entropy values indicate that, on average, the long wavelength range is most informative, followed by a mid-wavelength region between 400 nm and 500 nm. Interestingly, in a range where spectral information increases (490 nm and above), Rh1 is most sensitive, while the other available receptors are rather insensitive (Fig 2b). Together with the assessment of the power spectral density distribution of natural reflectance spectra, which argue in favor of including a fifth receptor type, this supports our conclusions from the model results.

Analysis of mutual information indicates that that Rh1 is the second most informative Drosophila opsin (see Fig 6b). In general, there is a trend that the more sensitive a receptor type is for the long wavelength the higher its mutual information. The differences of informational value in the four receptor systems are not very large (see Fig 6c), and the addition of Rh1 to the traditional four-receptor system increases the information by only 7%. Compared to the information added when going from three to four receptors, this seems not particularly large. However, it is substantial considering that the fifth principal component accounts for only 3% of the variability.

The important aspect is that Rh1 contributes information, considering that Rh1 is highly correlated with both Rh5 and Rh6.

This does not need to be an optimization and could, as suggested by Kelber and Henze [41], be due to convergence in visual pathways that intersect at higher levels, subserving other functions than optimizing for color vision.

On the other hand, it is possible that not all of the inner receptors contribute to color vision in Drosophila. It is known that the butterfly *Papilio*, with eight different opsins, is only tetrachromatic [30]. Our analysis of the wavelength discrimination data demonstrate that, at least in the visual range, just three receptors are best to model the data. Adding more receptors reduces goodness of fits, not only because of the higher number of parameters, but because discrimination increased where the data suggested poor discrimination.

Potentially, further opsins could contribute in the UV, where currently no wavelength discrimination data for Drosophila are available. However, the reflectance data indicate that among the four opsins there is not much variability in the mutual information, which would speak in favor of a contribution by Rh1 rather than one of the other opsins.

Concerning possible implementations it has been shown that the outer photoreceptors do not terminate in the medulla as the inner photoreceptors but in the lamina neuropil. From there three lamina monopolar cells (L1,L2,L3) connect directly to the medulla, where signals from outer and inner receptors converge [42, 43]. Interestingly, blocking the laminar monopolar cells L1–L3 inhibits blue/green discrimination in Drosophila [16]. Non-columnar projection neurons could mediate interommatidial combination of inner receptor signals [44], however, such combination would not be predicted by our best fitting model. In the calculation of the mutual information we have assumed the same noise level for all opsins. This is certainly an oversimplification, especially considering that there are more outer receptors with Rh1 than inner receptors, and more than twice as many pale than yellow ommatidia [5, 45]. While, in a receptor noise limited regime, this would have an effect on the information for the individual opsins, the values for systems combining several opsins will practically not change. As reported above, for combinations of four opsins there is already very little difference in the mutual information. This is mainly due to the spectral correlation structure between the spectral sensitivities and the smoothness of natural reflectances. Neither the high correlation between the shapes of the spectral sensitivities nor the smoothness of natural spectra, however, is changed by different noise levels when calculating mutual information.

Koshitake et al. [30] modeled wavelength discrimination in the butterfly with an approach that also builds on chromatic comparisons, but assumes that discrimination is limited by the noise in the photoreceptors. We found such a model to work poorly for the Drosophila data used here (data not shown). First, absolute thresholds could not be replicated, and even after introduction of a scaling parameter, fits with noise levels based on receptor count did not provide acceptable errors (p< 0.001).This could be an indication that color discrimination in Drosophila is not limited by receptor noise but by postreceptoral stages.

Troje [12] studied wavelength discrimination in the goldfly and found that it is possible to explain the behavioral data without the incorporation of the signals from Rh1. There are several prominent and also subtle differences between the data used in that study and the data we used. Like the data from Hernandez de Salomon and Spatz [24], the results by Troje [12] indicate good discrimination around 500 nm. Besides the difference that the model by Troje [12] tries to predict the learning curves directly, whereas our models predict the wavelength discrimination function, given the similarities in the data, why do the results differ?

The critical difference lies in the spectral sensitivity functions used. In particular the Rh6 spectral sensitivity function that we used, which was directly measured in Drosophila [14],however expressed in the outer receptors, is broader than the one used by Troje [12], which was measured in Musca [46] and is narrower because of screening by the R7 receptor. This Rh6 function has an absolute slope change in the region between 470 nm and 500 nm, and it is likely that with the Drosophila Rh6 spectral sensitivities used by us the results by Troje [12] would have been different. A different Rh6 spectral sensitivity could be an alternative explanation to a contribution of Rh1 to Drosophila wavelength discrimination, if the Rh6 curve would have a higher slope near 500 nm than near 470 nm. However, for this to occur the point of inflection of the Rh6 spectral sensitivity curve would need to be shifted by almost 50 nm, which seems unlikely with shielding from R7.

In conclusion, we have confirmed that the behavioral data on wavelength discrimination in Drosophila can hardly be understood without incorporating Rh1. This result is mainly due to the good discrimination around 500 nm which directly relates to the spectral sensitivity of the Rh1 opsin. Neither a different weighting scheme nor reasonable modifications in the derivation of the discrimination function alter this conclusion. The contribution of Rh1 to the best fitting models is prominent, and a model comparing signals from Rh1, Rh6 and Rh4 already provides a good explanation of the discrimination behavior of the wild type fly. With respect to the encoding of natural reflectance spectra, the spectral positioning of Rh1 is actually not optimal for discrimination. The power spectral density of natural spectra indicates that five receptor spectral sensitivities would be optimal for the visual range of Drosophila. However, this argument is based on the assumption of an equidistant sampling of the spectrum with rather broad receptoral functions located roughly fifty nanometer apart. It is rather obvious that this is not the case for Rh1, Rh3, and Rh4. Furthermore, the double peaked nature of the sensitivity of the Rh1 containing receptors render them suboptimal for unambiguous spectral discrimination [12]. Whether color vision is important for Drosophila at all is an open question [47], but the behavioral data indicate a role for Rh1 when color discrimination is tested, and our theoretical analysis of natural reflectance data shows that there is information in the signals of a fifth opsin in general, and Rh1 in particular.

## Supporting Information

S1 Data Spectra IDs. S1_Data.csv lists the ids (see http://www.reflectance.co.uk/) of all spectra used in this study.
(CSV)
